# Robust light field angular super-resolution via multi-dimensional feature fusion and attention-guided refinement

**DOI:** 10.1371/journal.pone.0353845

**Published:** 2026-07-15

**Authors:** Xiyao Hua, Boni Su, Daili Yang

**Affiliations:** School of Big Data and Artificial Intelligence, Chengdu Technological University, Chengdu, Chinax; Communication University of Zhejiang, CHINA

## Abstract

Angular super-resolution (ASR) is a fundamental task in light field (LF) imaging, aimed at reconstructing a dense LF from sparsely sampled views. Despite significant progress, current methods often struggle to preserve consistency in complex scenarios such as severe occlusions and large-disparity regions. In this paper, we propose a robust attention-guided multi-dimensional feature fusion network (LFAMF) for LF angular reconstruction. The proposed framework comprises two synergistic stages: a multi-dimensional feature fusion stage and an attention-guided refinement stage. Specifically, we design a multi-stream subnetwork (MFNet) to extract intrinsic physical characteristics across the spatial, angular, EPI, and pseudo-video sequence domains. Simultaneously, a geometry-prior-based subnetwork (GSPNet) is incorporated to leverage scene structure for improved texture preservation. To effectively integrate these complementary streams, an attention-guided fusion subnetwork (AFNet) is employed to adaptively merge intermediate results. Extensive experiments on both synthetic and real-world datasets demonstrate that the LFAMF model significantly outperforms state-of-the-art methods, particularly in maintaining structural integrity at occlusion boundaries and highly textured areas while ensuring superior angular consistency.

## Introduction

Light field (LF) imaging has emerged as a powerful modality in computer vision. By capturing both the spatial intensity and angular direction of light rays, it provides a comprehensive representation of scene geometry. This capability facilitates various downstream applications, such as saliency detection [1], post-capture refocusing [2], and 3D reconstruction [3]. However, the intrinsic hardware trade-off between spatial and angular resolution in mainstream LF cameras (e.g., microlens-based or camera arrays) limits the acquisition of high-density LFs. Consequently, light field angular super-resolution (LFASR), which aims to synthesize dense, high-quality views from a sparse set of inputs, has emerged as a pivotal research challenge.

Existing LFASR methods can be broadly categorized into non-depth-based and depth-based methods. Non-depth-based methods [4–6] typically leverage deep convolutional neural networks (CNNs) to implicitly learn the mapping between sparse and dense views without explicit geometric modeling. Although these methods excel at recovering fine textures in simple scenes, they often struggle to capture the long-range geometric constraints of the 4D LF, leading to blurring or ghosting artifacts in large-disparity regions. In contrast, depth-based methods [7–9] explicitly estimate the disparity information to warp input views for reconstruction. However, their performance is inherently dependent on the quality of the estimated depth maps. In complex scenarios involving severe occlusions or non-Lambertian surfaces, depth estimation often becomes unreliable, leading to significant distortions or discontinuities in the reconstructed pixels. In this paper, we argue that the limitations of these two paradigms are fundamentally complementary. While non-depth-based feature representations provide robustness to local intensity variations, depth-based geometric priors offer the structural backbone necessary for maintaining angular consistency. To bridge this gap, we propose the light field attention-guided multi-dimensional feature fusion network (LFAMF), a robust framework designed for high-fidelity LF reconstruction in complex scenes. The core philosophy of LFAMF lies in its hierarchical fusion strategy, which synergistically explores the intrinsic characteristics of the LF across multiple domains.

The proposed LFAMF consists of two stages: multi-dimensional feature fusion and attention-guided refinement. In the first stage, we design a multi-stream subnetwork (MFNet) to extract features from the spatial, angular, epipolar plane image (EPI), and pseudo-video sequence (PVS) domains. These diverse representations capture both the slope-consistency of EPIs and the inter-view motion cues of the PVS. Simultaneously, a depth-based subnetwork (GSPNet) is incorporated to leverage scene geometry through an improved encoder-decoder architecture with an expanded receptive field. In the second stage, an attention-guided fusion subnetwork (AFNet) is introduced to adaptively merge these complementary streams. Unlike simple concatenation, AFNet acts as a quality evaluator, assigning higher confidence to reliable depth-based projections while utilizing robust non-depth features to compensate for occluded or textureless regions.

The main contribution of this paper can be summarized as follows:

(1) We propose LFAMF, which effectively bridges non-depth-based feature learning and depth-based geometric priors. By treating LF reconstruction as a multi-domain fusion problem, the model achieves unprecedented robustness in challenging scenarios such as large disparities and dense occlusions.
(2) We introduce MFNet and GSPNet to comprehensively model the 4D LF structure. By integrating PVS and EPI features with high-precision geometric priors from an improved depth estimation module, the network captures complex inter-view correlations and preserves the inherent disparity structure.
(3) We design AFNet to dynamically balance the strengths of non-depth-based and depth-based streams. This mechanism effectively mitigates the occlusion fragility inherent in geometry-dependent models, ensuring superior angular consistency and pixel-wise fidelity.

The rest of this paper is organized as follows. The Related Works section reviews the related work. The Motivation and Occlusion Analysis section analyzes the limitations and complementary characteristics of depth-based and non-depth-based methods. The Proposed Method section details the proposed LFAMF architecture. The Experimental Results section presents the experimental results and analysis, and the Conclusion section concludes the paper.

## Related works

Light field angular super-resolution (LFASR), also known as LF reconstruction or view synthesis, refers to the process of generating novel views from the given views. Existing methods can be roughly categorized into depth-based methods and non-depth-based methods, depending on whether they leverage explicit depth information.

### Depth-based methods

Depth-based methods commonly adopt a two-stage paradigm: they first estimate the scene disparity or depth, and then synthesize novel views by warping the input views to the desired positions. Wanner and Goldluecke [10] first used structure tensors for depth map estimation, and then formulated the subsequent view synthesis as a complex optimization problem. Mitra and Veeraraghavan [2], utilized a Gaussian Mixture Model (GMM) to model LF patches, reconstructing them after depth estimation. Pearson et al. [11] introduced a layer-based model, utilizing multiple depth layers to represent scene geometry and enabling view synthesis at arbitrary angular positions. Zhang et al. [12] exploited a phase-based method leveraging a micro-baseline stereo pair for robust 4D LF reconstruction, while Zhang et al. [13] introduced a patch-based decomposition approach that synthesized images on multiple layers derived from the central view. Despite these advancements, traditional depth estimation methods consistently struggled with sparse LF inputs, often producing aliasing effects at occlusion boundaries.

With the rapid development of deep learning, researchers have made efforts to apply deep neural networks to reconstruct LF. Specifically, Kalantari et al. [7] made the first attempt to use CNN for densely-sampled LF reconstruction. Their framework decomposed novel view synthesis into disparity estimation and subsequent color estimation. However, as each novel view is independent predicted within their architecture, this approach inherently neglected the angular correlations and photo-consistency. Jin et al. [8] proposed a novel end-to-end network for LF reconstruction based on depth information. Their network consists of depth estimation, physically-based view warping, and an LF blending module that aggregates the contributions from all warped views via spatial-angular interleaved convolution. This explicit geometric modeling enables their method to achieve superior performance, particularly for large baseline LFs. Later, they [14] further introduced a coarse-to-fine approach for densely-sampled LF reconstruction, where 4D disparity maps were initially estimated and confidence maps were utilized to evaluate and merge the warped views. Shi et al. [15] proposed a structure-dependent network that fuses reconstruction results at both the pixel level and the feature level. Their method first estimates disparity maps using an optical flow network. Subsequently, both the input views and their high-level VGG features are warped to the target positions. The final views are synthesized by merging the two-level warped outputs using an adaptive soft combination mask. Ko et al. [16] addressed both angular and spatial SR by proposing an LF reconstruction network that utilizes a trainable disparity estimator. This estimator is specifically used to obtain disparity-compensated multi-view features. These aligned features are then processed and merged by an adaptive feature remixing module. Liu et al. [17] proposed a two-stage geometry-assisted multi-representation network for LFASR, which fuses structural features of multiple LF representations and intra-inter LF information to achieve superior reconstruction performance on real and synthetic scenes. Recently, Cai et al. [9] proposed a disparity enhancement network based on morphological filtering to address replication errors in edge regions during large disparity LF reconstruction. The above depth-based methods heavily depend on the quality of estimated depth, which makes them difficult to non-Lambertian surfaces and small disparity scenes.

### Non-depth based methods

The non-depth-based methods usually do not require explicit depth information, they instead treat LF reconstruction as approximating the plenoptic function, learning a direct mapping from sparse to dense LF images. These methods encompass traditional algorithms and subsequent CNN-based models. Early traditional methods utilized domain transforms and signal priors. Shi et al. [18] formulated reconstruction as a sparsity optimization problem in the continuous fourier domain, often leveraging only boundary views as input. Alternatively, Vagharshakyan et al. [19] employed an iterative regularization algorithm based on the sparse representation of EPIs in the discrete shearlet transform domain. Furthermore, some studies address compressive sensing in LF photography. Marwah et al. [20] proposed a camera architecture that utilizes overcomplete dictionaries for LF reconstruction. To enhance efficiency, Kamal et al. [21] later reduced the computational cost for dictionary learning by exploiting a joint tensor low-rank and sparse prior.

Recently, owing to their robust representation and generalization capabilities, CNN-based methods have emerged as the dominant paradigm. For example, Yoon et al. [22,23] pioneered a CNN-based model that jointly super-resolved the LF in both the spatial and angular domains. Their architecture first concatenated spatially super-resolved SAIs, and then passed them through a second CNN for angular SR. However, their approach was specifically designed for scale 2 angular SR, limiting its flexibility for adaptation to more sparsely sampled LF inputs. Wu et al. [24,25] proposed a “blur-restoration-deblur” framework to address the information asymmetry between the spatial and angular dimensions of sparse LFs. This strategy involved designing a 1D blur kernel to process EPIs, with the network trained to super-resolve the blurred features. Although the method’s effectiveness was validated in the fourier domain, it failed to robustly handle large disparities. Later, Wu et al. [26] further introduced a framework based on sheared EPIs. This method leverages the observation that an EPI exhibits a clear structure when sheared according to its disparity value. Specifically, low angular resolution EPIs are first sheared with different disparities and then upsampled. The resulting upsampled EPIs, generated through various shearing transformations, are finally combined via a CNN using learned fusion scores. Yeung et al. [27] proposed a network to reconstruct LFs by utilizing spatial-angular clues. They approximated 4D convolutions using spatial-angular alternating convolutions and synthesized novel views within the channel dimension. While their method offered a fast inference speed, a key limitation was its difficulty in handling large-disparity scenes. To address the bottleneck of limited angular resolution in plenoptic cameras, Wang et al. [28] proposed an end-to-end framework with a Pseudo 4DCNN, which assembled from stacked EPI-based 2D strided convolutions and angular conversion-connected 3D detail-restoration CNNs. Liu et al. [4] leveraged multi-angular epipolar geometry to enhance LF reconstruction. Their approach fused input views stacked from different directional EPIs to better learn the mapping from sparse to dense LFs. Zhang et al. [5] grouped LFs into micro-lens and view image stacks, then employed 3D CNNs for feature extraction and deconvolution layers to upsample micro-lens images. Meng et al. [29] proposed a 4D-convolution-based HD-DRNet for LF spatial-angular SR, integrating view-group normalization, stage-wise loss, and multi-range training to boost performance. Hu et al. [6] utilized dense skip connections to efficiently model correlation information in a way that respects the domain asymmetry between spatial and angular dimensions. Wu et al. [30] introduced the non-local attention mechanism to the LF reconstruction domain via their spatial-angular attention network. Very recently, to solve the non-Lambertian effect and large disparity issues, Liu et al. [31] proposed an efficient angular super-resolution network called EASR. EASR extracts multi-scale spatial-angular features from sparse SAIs via simple 3D Unet and conducts angular super-resolution on macro-pixel features, which enhances its ability to handle large disparities. Wang et al. [32] proposed a generic feature disentangling mechanism with domain-specific convolutions to separate LF spatial-angular information, and developed three task-specific networks for spatial SR, angular SR, and disparity estimation. Hua et al. [33] proposed a transformer-based network (LFRTR) for dense LF reconstruction, which integrates angular and spatial transformers to capture inter-view angular correlations and intra-view spatial information. It also incorporates image and angular dense skip connections to enhance information flow. Liu et al. [34] developed an LFASR network integrating a global-local coupled convolutional transformer network, a deep deblurring network, and a texture-aware feature fusion network. Later, they [35] presented an implicit and detail-enhanced network (IDNet) for LF spatial-angular SR, leveraging 3D convolution for joint feature extraction. Mao et al. [36] proposed a baseline method with an artifact-aware loss function and constructed a multi-view dataset for the sparse-to-dense inbetweening task of LF dense intermediate view reconstruction.

In summary, while both depth-based and non-depth-based methods have demonstrated significant potential, a unified framework that effectively synergizes the feature robustness of non-depth-based learning with the geometric precision of depth-based priors is still lacking. Most existing approaches struggle to balance these two paradigms, particularly when facing complex occlusions and large disparities. This limitation serves as the primary motivation for our proposed LFAMF, which aims to bridge this gap through a multi-dimensional feature fusion and attention-guided refinement strategy.

## Motivation and occlusion analysis

### Limitations of existing LFASR methods

Existing LFASR methods suffer from intrinsic limitations, making it hard to apply them robustly in scenes with mixed complexity (e.g., simultaneous large disparity and complex occlusion). We summarize the shortcomings of these two main categories:

(1) *Depth-based methods*. Reconstruction approaches relying on explicit depth information [7,8] estimate the scene depth map and then warp input views to synthesize novel viewpoints. This two-stage process critically depends on the precision of the estimated depth. Inaccurate disparity values, particularly at occlusion boundaries or on non-Lambertian surfaces, inevitably introduce severe geometric distortion and ghosting artifacts.
(2) *Non-depth-based methods*. Methods that bypass explicit depth estimation [4,6,27] implicitly learn the scene structure to establish a direct mapping for view synthesis. However, due to the limited receptive field of these networks and the absence of explicit geometric guidance, they often fail to capture long-range correlation necessary for handling large disparity scenes, leading to structural blurring and a loss of high-frequency details.

**Fig 1:**
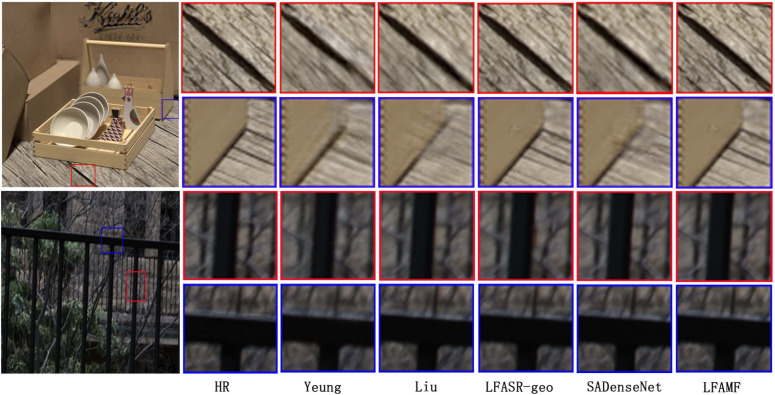
Visual comparison of reconstruction results between two types of methods across different scenes. The scenes are selected from the HCInew [38] and Occlusions [37] datasets. All data were used in accordance with their respective CC BY 4.0 licenses, and all output images were produced by the authors.

To rigorously demonstrate these complementary failure modes, we conduct a visual analysis of representative methods. As shown in Fig 1, non-depth-based models (e.g., Yeung [27], Liu [4], and SADenseNet [6]) exhibit perceptible blurring in high-frequency regions under large-disparity conditions (Row 1), such as the wooden board textures. Conversely, the depth-based LFSR-geo [8] suffers from severe artifacts at occlusion boundaries (Row 2), particularly along the fence edges.

**Fig 2:**
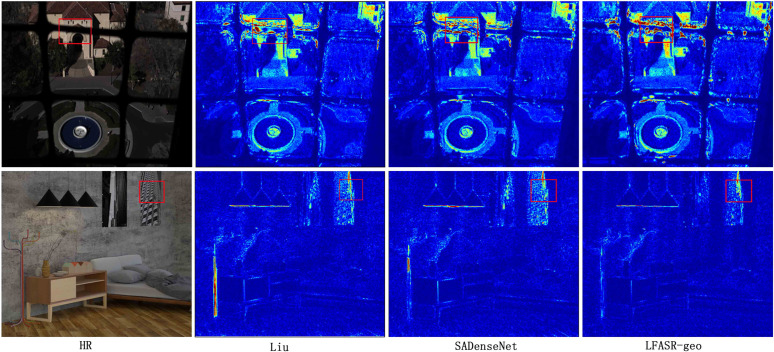
Reconstruction results of Liu [4], SADenseNet [6], and LFASR-geo [8] across different scenes from the HCInew [38] and Occlusions [37] datasets. (All results are rendered by the authors under the same CC BY license mentioned in Fig 1).

Furthermore, the error map visualization in Fig 2 reinforces this observation. In occlusion-heavy regions (Scene 1), the depth-based LFASR-geo [8] struggles to accurately reconstruct the window panes (red box), while non-depth methods (Liu [4] and SADenseNet [6]) yield relatively complete outlines. In non-occluded large-disparity areas (Scene 2), the situation reverses: depth-based methods successfully project intricate textures (e.g., wall details) guided by reliable geometric priors, while non-depth methods fail to recover such complex patterns.

These reconstruction instances unequivocally illustrate that the two existing classes of LFASR methods cannot simultaneously address complex scenes involving both occlusion and large disparity. Crucially, their respective strengths and weaknesses demonstrate a significant degree of complementarity, suggesting that a rational and effective combination of the two paradigms is necessary to leverage their advantages and globally enhance LFASR performance. The core conceptual insight of this work is that LFASR should not be treated as a single-paradigm task; rather, it requires a hybrid architecture where implicit multi-dimensional features (providing texture consistency) and explicit geometric priors (providing structural constraints) are treated as synergistic yet potentially conflicting streams that must be dynamically reconciled.

### Light field occlusion analysis

**Fig 3:**
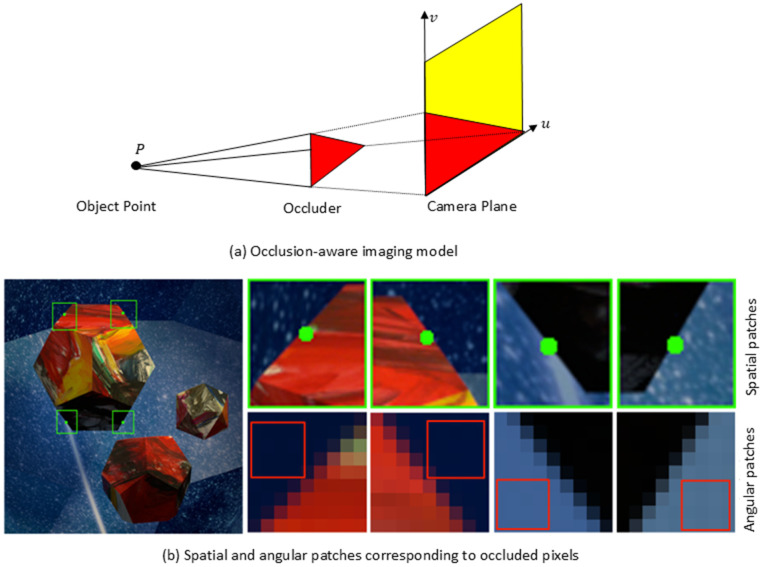
Schematic diagram of occlusion model.

Occlusion remains a fundamental bottleneck in LF reconstruction. Under the ideal Lambertian assumption and non-occlusion conditions, light rays originating from a single scene point should maintain photo-consistency across all views. When refocused to the correct depth, these viewpoints converge to a single point, satisfying angular consistency. However, occlusion breaks this consistency, leading to unreliable depth estimation and subsequent artifacts near depth discontinuities. To formalize this, we define the angular patch as:


$$\begin{array}{c} A_{d}^{p}(u,v)=I(u,v,x+(u_{c}-u)d,(v_{c}-v)d), \end{array}$$ (1)

where $A_{d}^{p}$ denotes the angular patch at spatial location *p*(*x*,*y*) with a disparity (depth) value of *d*, while $u_{c}$ and $v_{c}$ are the coordinates of the central view. This angular patch comprises the corresponding pixels from all sub-aperture images refocused to depth *d*. Fig 3(a) illustrates the imaging model under occlusion. Due to an occluding object, a scene point *P* is only captured by cameras in the yellow angular region, while the red region records the occluder’s intensity. Consequently, the angular patch exhibits a bimodal or non-uniform distribution. Fig 3(b) presents four occlusion scenarios and their corresponding refocused angular patches. A crucial observation is that while the entire patch may violate the angular consistency constraint, a subset of the patch—specifically, at least one of its subdivisions—often maintains consistency (as indicated by the red boxes).

This analysis provides a vital insight: when occlusion occurs, certain off-center views often remain unoccluded relative to the target point. This suggests that by adaptively re-weighting different views based on their occlusion status, the errors induced by depth discontinuities can be effectively suppressed. Beyond a simple parallel combination of modules, our fundamental advance lies in acknowledging the inherent uncertainty of geometry-based warping in these regions. While explicit geometry (GSPNet) offers a “global skeleton” for reconstruction, it is prone to artifacts where the angular consistency is broken. By introducing an attention-guided fusion mechanism (AFNet) as a “dynamic arbitrator,” our framework learns to shift reliance toward implicit multi-representation features (MFNet) in occluded or non-Lambertian areas while enforcing geometric rigor elsewhere. This physical insight serves as the theoretical foundation for our attention-guided fusion subnetwork (AFNet), which learns to dynamically balance the contributions of multi-dimensional features and geometric priors to achieve occlusion-robust reconstruction.

## Proposed method

**Fig 4:**
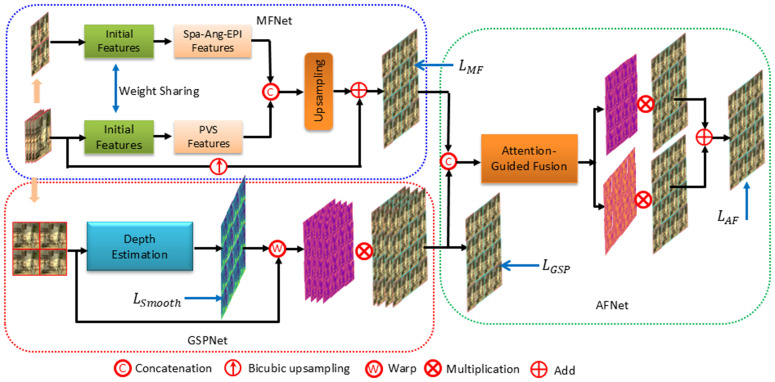
Detailed architectural overview of the proposed LFAMF network. The joint optimization pipeline flows horizontally and is organized into three distinct structural blocks: (a) The Top Block (MFNet) consists of multi-stream parallel branches designed to capture rich complementary physical characteristics from spatial, angular, EPI, and pseudo-video sequence domains; (b) The Middle Block (GSPNet) serves as the geometry-prior stream that executes disparity estimation and view warping to maintain macro-structural awareness; and (c) The Right Block (AFNet) adaptively aggregates these intermediate features through attention-guided fusion and residual refinement blocks to synthesize the final robust dense LF.

As illustrated in Fig 4, the proposed light field attention-guided multi-dimensional fusion network (LFAMF) consists of three primary components: the multi-dimensional feature fusion subnetwork (MFNet), the geometric structure prior subnetwork (GSPNet), and the attention-guided fusion subnetwork (AFNet). The LFAMF synergistically integrates multi-domain features and explicit geometric priors to achieve robust reconstruction in complex scenarios.

### Multi-dimensional feature fusion subnetwork

**Fig 5:**
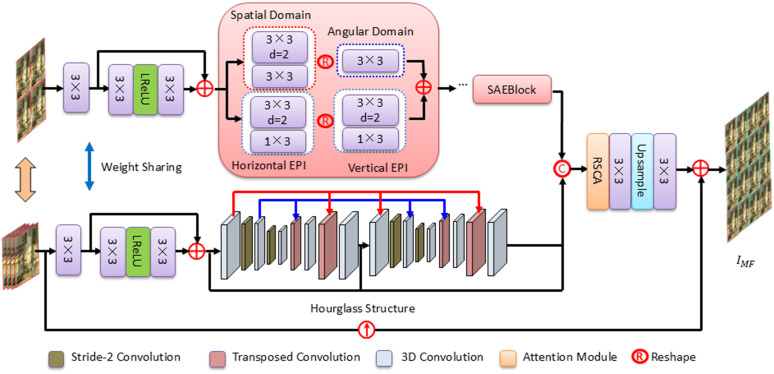
The architecture of MFNet.

For the task of dense reconstruction from sparse LF images, effectively exploiting the intrinsic multi-dimensional physical characteristics of the 4D LF is crucial for enhancing reconstruction performance. To this end, the LFAMF model incorporates a multi-dimensional feature fusion subnetwork (MFNet). As illustrated in Fig 5, the MFNet architecture comprises two parallel branches. The top branch utilizes the spatial-angular-epipolar block (SAEBlock) to explore intra-view contextual information and inter-view complementarity. Simultaneously, the bottom branch transforms the LF into a pseudo-video sequence (PVS) and employs 3D convolutions to aggregate spatio-angular correlations.

In the MFNet pipeline, the input LF is first mapped into a high-dimensional feature space through a weight-sharing 2D convolutional layer and residual blocks. Subsequently, multi-dimensional features are aggregated via multiple cascaded SAEBlocks integrated within an hourglass structure. To optimize the integration of these features, the residual spatial-channel attention (RSCA) module is utilized to recalibrate the feature maps, ensuring the network prioritizes the most salient information in the feature space. Finally, the extracted LF features are upsampled from the spatial domain to the angular domain to reconstruct the high-angular-resolution LF. The SAEBlock decomposes the 4D LF into various subspace domains and extracts domain-specific features using 2D convolutions. Within the hourglass structure, two 3×3×3 convolutions with a stride of (1, 2, 2) are applied for spatial downsampling to enlarge the receptive field and aggregate more spatial context. Skip connections are established between corresponding layers to facilitate information flow between shallow and deep features. To maintain high angular consistency, angular upsampling is implemented using sub-pixel convolutions (for 2×2–8×8 tasks) or 3D transposed convolutions (for 2×2–7×7 tasks). Furthermore, MFNet adopts a global residual connection, which enables the backbone to focus on learning the residual mapping between the low-resolution and high-resolution LFs, thereby reducing the learning complexity of the network.

### Geometric structure prior subnetwork

To enhance the reconstruction quality in scenarios with large disparities, we propose the geometric structure prior subnetwork (GSPNet). As illustrated in Fig 4, the GSPNet architecture consists of three functional modules: depth estimation, view warping (projection), and attention-guided fusion. Specifically, the depth estimation module first predicts the disparity maps for each input view. Subsequently, based on the estimated 4D depth information, each input view is warped to the target positions to synthesize an initial set of reconstructed LF views.

However, distortions in these initial reconstructions are inevitable due to depth estimation inaccuracies, non-Lambertian surfaces, and occlusions. Based on the occlusion analysis in the Motivation and Occlusion Analysis section, even when occlusions occur, at least one of the four corner input views typically remains unaffected. Leveraging this insight, GSPNet employs an attention-based fusion strategy to address occlusion-related artifacts. By utilizing a softmax-normalized attention mechanism, the network generates a weight matrix for the synthesized views, which guides the fusion process to produce the final geometrically consistent reconstruction.

(1) *Depth estimation module*. Depth estimation is a dense prediction task that aims to assign a disparity value to each pixel of the input views. While previous methods like LFASR-geo [8] utilize standard CNNs for depth prediction, their architectures often suffer from a limited receptive field, making it challenging to capture long-range dependencies within the scene. Consequently, the resulting depth maps may lack the accuracy required for high-fidelity reconstruction.

**Fig 6:**
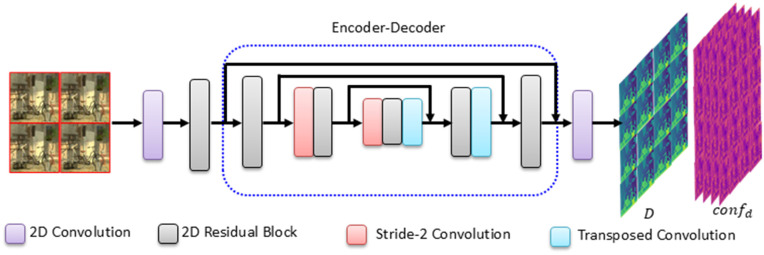
The architecture of depth estimation module.

To address these limitations, we design an Encoder-Decoder based depth estimation module. As shown in Fig 6, this module incorporates two levels of downsampling and upsampling, integrated with skip connections. The downsampling operations effectively enlarge the spatial receptive field while reducing computational complexity. Meanwhile, the skip connections facilitate the propagation of fine-grained spatial details from shallow layers to deeper ones, enhancing feature reuse and information flow across scales.

In our implementation, downsampling and upsampling are achieved using stride-2 convolutions and transposed convolutions, respectively. To optimize the model’s parameter efficiency, we employ channel concatenation for skip connections in the shallower layers, while utilizing element-wise addition in the deeper layers. By extracting multi-scale features and spatial correlations, this module is capable of capturing rich semantic information and handling a relatively large range of disparities. The entire depth estimation process can be formulated as:


$$\begin{array}{c} D,conf_{d}=f_{d}(I_{LR}(u^{\prime },v^{\prime },x,y)), \end{array}$$ (2)

where *D* denotes the estimated 4D disparity map, and $conf_{d}$ represents its corresponding attention (or confidence) map.

(2) *View warping module*. Based on the disparity map *D* predicted by the depth estimation module, GSPNet projects the input views to target positions to synthesize novel views. This view projection process (backward warping) is described as:


$$\begin{array}{c} W=f_{w}(I_{LR}(u^{\prime },v^{\prime },x,y),D), \end{array}$$ (3)

where *W* denotes the synthesized novel views.

(3) *Attention-guided fusion module*. For the initial set of reconstructed LF images generated by projecting the four corner input views, the LFAMF network utilizes the LF occlusion model and an attention-guided fusion strategy. This approach assigns adaptive weights to different projected views and performs a weighted summation to generate the fused LF image. The fusion process is defined as:


$$\begin{array}{c} conf_{d}^{\prime }=Softmax(conf_{d}), \end{array}$$ (4)

$$\begin{array}{c} I_{GSP}=\sum_{u^{\prime },v^{\prime }}conf_{d}^{\prime }(u^{\prime },v^{\prime },x,y)⊙W(u^{\prime },v^{\prime },x,y), \end{array}$$ (5)

where $I_{GSP}$ is the final fused LF image, and ⊙ denotes the element-wise multiplication.

**Fig 7:**
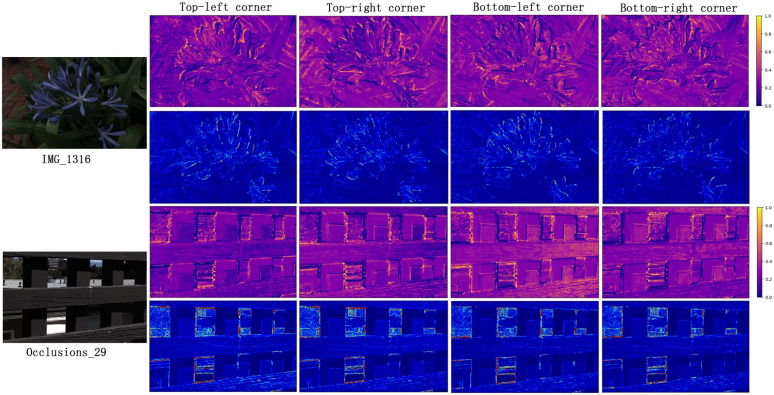
Attention maps and error maps corresponding to the four input views across different scenes from the HCInew [38] and Occlusions [37] datasets. (All results are rendered by the authors under the same CC BY license mentioned in Fig 1).

Fig 7 illustrates the attention maps and the corresponding error maps for the LF images generated from the four input views. It is observed that for novel views synthesized from different source views, the errors around occlusion boundaries are closely related to the spatial position of the source view. The learned attention maps effectively identify these error-prone regions in each synthesized view, providing precise guidance for the fusion process. Specifically, non-occluded views are assigned higher weights (indicated by yellow regions), while occluded views receive lower weights (indicated by blue regions), which is consistent with the angular block results from the occlusion model analysis in the Motivation and Occlusion Analysis section. This attention-guided fusion strategy effectively suppresses reconstruction artifacts caused by occlusions, thereby significantly improving the overall reconstruction performance.

### Attention-guided fusion subnetwork

**Fig 8:**
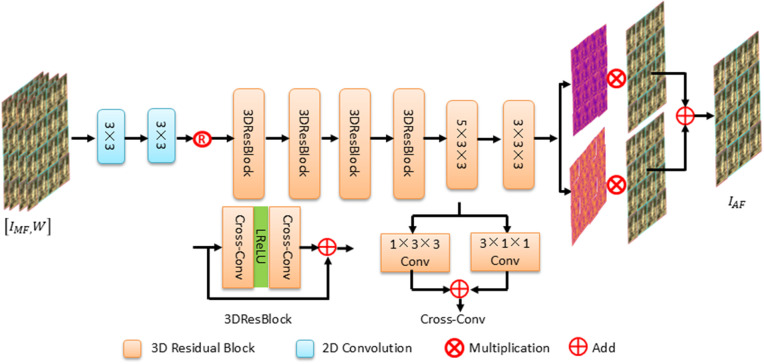
The architecture of AFNet.


As discussed previously, MFNet extracts the intrinsic multi-dimensional physical characteristics of the LF from the spatial, angular, EPI, and pseudo-video sequence (PVS) domains. By upsampling the sparse input LF directly in the angular domain without explicit depth estimation, MFNet effectively bypasses the challenges associated with occlusions and non-Lambertian surfaces. Conversely, GSPNet leverages the explicit geometric structure of the LF to project input views to target positions, thereby preserving intricate textures and fine-grained details via geometric priors. Given that these two subnetworks exhibit strong complementarity, their integration allows the model to capitalize on their respective strengths, significantly enhancing the representation and modeling capabilities for complex scenes involving large disparities and severe occlusions. To this end, the LFAMF model incorporates an attention-guided fusion subnetwork (AFNet) to effectively fuse the intermediate results from both branches. As illustrated in Fig 8, the AFNet architecture consists of two 2D 3×3 convolutional layers, four 3D residual blocks, and an attention-guided fusion module. The 2D convolutions are employed to extract high-level spatial features from the intermediate results of both subnetworks. The four 3D residual blocks are designed to fuse the inter-view and intra-view spatio-geometric structures along with multi-dimensional features. Within the attention-guided fusion module, two 3D convolutions of different kernels are first utilized to progressively compress the angular dimension. Subsequently, a Softmax function generates the attention maps, which are used to perform a weighted summation for the final high-resolution LF reconstruction. To enhance computational efficiency and reduce the parameter count, we utilize cross-convolutions (as depicted in the figure) as a lightweight alternative to standard 3D convolutions. The entire fusion process can be formulated as follows:

$$\begin{array}{c} conf_{w},conf_{MF},I_{GSP}^{\prime },I_{MF}^{\prime }=f_{AF}\left(\left[I_{MF},W\right]\right), \end{array}$$ (6)

$$\begin{array}{c} conf_{w}^{\prime },conf_{MF}^{\prime }=Softmax\left(\left[conf_{w},conf_{MF}\right]\right), \end{array}$$ (7)

$$\begin{array}{c} I_{AF}=conf_{w}^{\prime }⊙I_{GSP}^{\prime }+conf_{MF}^{\prime }⊙I_{MF}^{\prime }, \end{array}$$ (8)

where $conf_{w}^{\prime }$ and $conf_{MF}^{\prime }$ represent the learned attention maps corresponding to the MFNet and GSPNet branches, respectively; $I_{AF}$ denotes the final reconstructed high-resolution LF image; and [·] signifies the channel-wise concatenation operation.

### Loss functions

The LFAMF model is designed to reconstruct a dense LF from a given sparsely sampled input. To facilitate effective training, the L1 loss function is employed to supervise the intermediate outputs, $I_{MF}$ and $I_{GSP}$, as well as the final reconstructed LF image, $I_{HR}$. The objective is to minimize the mean absolute error (MAE) between these predicted results and their corresponding ground truth (GT) labels:


$$\begin{array}{c} L_{MF}=\sum_{u,v,x,y}\left(\left|I_{MF}(u,v,x,y)-I_{HR}(u,v,x,y)\right|\right), \end{array}$$ (9)

$$\begin{array}{c} L_{GSP}=\sum_{u,v,x,y}\left(\left|I_{GSP}(u,v,x,y)-I_{HR}(u,v,x,y)\right|\right), \end{array}$$ (10)

$$\begin{array}{c} L_{AF}=\sum_{u,v,x,y}\left(\left|I_{AF}(u,v,x,y)-I_{HR}(u,v,x,y)\right|\right) \end{array}$$ (11)

Since high-precision ground truth depth maps for real-world scenes are often difficult to obtain, the depth estimation module within the GSPNet subnetwork is trained in an unsupervised manner. To enhance the structural accuracy and spatial coherence of the estimated disparity maps, a smoothness loss based on second-order gradients is incorporated [8]. The smoothness loss function is defined as:


$$\begin{array}{c} L_{smooth}=\sum_{u,v,x,y}\left(\left|\nabla_{xx}D\right|+\left|\nabla_{xy}D\right|+\left|\nabla_{yy}D\right|+\left|\nabla_{yx}D\right|\right) \end{array}$$ (12)

where ∇ denotes the second-order gradient operator.

Collectively, the total loss function used for training the LFAMF network is defined as a weighted combination of the reconstruction and smoothness terms:


$$\begin{array}{c} L=L_{MF}+L_{GSP}+\lambda L_{Smooth}+L_{AF}, \end{array}$$ (13)

where λ represents the regularization coefficient for the smoothness loss, which is empirically set to 0.001 in our experiments.

In our implementation, the three reconstruction loss terms ($L_{MF}$, $L_{GSP}$, and $L_{AF}$) are assigned equal weights (i.e., 1:1:1). The rationale behind this uniform weighting strategy is twofold. First, from a numerical perspective, since all three losses are uniformly regularized under the L1-norm variant on normalized domains, their gradients naturally share a tightly aligned scale, which inherently averts gradient vanishing or dominance during joint optimization. Second, from a structural perspective, these terms supervise complementary dimensions of multi-dimensional feature alignment, geometric structure awareness, and final feature fusion, all of which are equally vital for addressing complex occlusions. This empirical equal-weight setup successfully aligns with prominent LF reconstruction paradigms, such as the geometry-aware multi-loss fusion proposed by Jin et al. [14], which verified that equal joint supervision guarantees robust convergence and superior visual quality without introducing redundant hyper-parameter tuning.

## Experimental results

In this section, we first introduce the datasets and experimental details. Then, we conduct a comprehensive quantitative and qualitative comparison with other state-of-the-art methods. Finally, an ablation study is performed to validate the effectiveness of the individual components of the LFAMF model and evaluate their respective impacts on the reconstruction performance.

### Datasets and implementation details

To evaluate the effectiveness of the proposed LFAMF model, extensive experiments were conducted on both real-world and synthetic datasets. The detailed allocation of scenes for training and testing is summarized in Table 1.

**Table 1:** Detailed data splitting and scene allocation for experiments.

Dataset Category	Source/Subset	Total Scenes	Training	Testing (Specific Scenes)
Synthetic	HCInew [38]	20 (Train)	20	–
	HCInew [38] & HCIold [39]	9 (Test)	–	Buddha, Buddha2, monasRoom, papillon, stillLife, bedroom, bicycle, dishes, herbs
Real-world	Kalantari [7]	100(Train)	100	–
	30Scenes [7]	30 (Test)	–	All 30 scenes
	Reflective [37]	15 (Test)	–	All 15 scenes
	Occlusions [37]	25 (Test)	–	All 25 scenes

(1) *Datasets*. The real-world data were captured using a Lytro Illum LF camera under natural lighting conditions. Specifically, the training set comprises 100 scenes provided by Kalantari [7], while 70 scenes selected from the 30Scenes [7] (30 scenes), Reflective [37] (15 scenes), and Occlusions [37] (25 scenes) datasets were used for testing. The Reflective and Occlusions datasets are particularly challenging due to the presence of non-Lambertian surfaces and complex occlusion boundaries, which significantly increase the difficulty of the reconstruction task.

Since real-world datasets typically feature a limited disparity range (within [−1, 1] pixels), we further evaluated the LFAMF model on synthetic datasets to analyze its robustness in large-disparity scenarios. For synthetic data, 20 scenes from the HCInew [38] dataset were used for training, while 9 scenes selected from HCInew(bedroom, bicycle, dishes and herbs) and HCIold [39](Buddha, Buddha2, monasRoom, papillon and stillLife) served as the test set. To mitigate the impact of optical distortion, we followed common practice and extracted the central 8×8 views for both training and testing.

(2) *Implementation details*. During the training phase, each sub-aperture image (SAI) was cropped into 64×64 patches with a stride of 16. To enrich the training samples and prevent overfitting, data augmentation techniques, including random flipping and 90-degree rotations, were applied. Our model was trained for two angular reconstruction tasks: 2×2–7×7 and 2×2–8×8 (i.e., reconstructing dense 7×7 or 8×8 LFs from a 2×2 sparse input).

In the MFNet subnetwork, the number of SAEBlocks was set to 4, and the feature channel dimension C was set to 48. For a fair comparison with state-of-the-art methods [4,6,8], the LFAMF model was trained on the Y-channel of the YCbCr color space. Performance was quantitatively assessed using peak signal-to-noise ratio (PSNR) and structural similarityindex (SSIM).

The network was optimized using the Adam optimizer with a batch size of 4. The initial learning rate was set to 2e-4 and was halved every 15 epochs. The total training duration was 70 epochs. We monitored the training and validation loss curves, observing that the loss reached a stable plateau after approximately 60 epochs, indicating full convergence. To prevent potential overfitting, we employed extensive data augmentation and weight decay regularization. The proposed model was implemented with PyTorch and was trained and tested on a PC with an NVIDIA RTX 3090Ti GPU.

### Comparison with state-of-the-art methods

In this section, we compare the proposed LFAMF model with nine state-of-the-art LF angular SR methods on both 2×2–7×7 and 2×2–8×8 reconstruction tasks. The competitors include three depth-based methods (Kalantari [7], LFASR-geo [8], and GAM [17]) and six learning-based methods that do not explicitly rely on depth information (ShearedEPI [26], Yeung [27], Liu [4], SADenseNet [6], LFRTR [33], and DistgASR [32]). For a fair comparison, all methods were retrained on the same datasets using their publicly available codes.

#### Evaluation on 2 × 2–7 × 7 reconstruction.

1 *Quantitative evaluation*.The quantitative results for the 2×2–7×7 task are summarized in Table 2, where the best and second-best results are highlighted in bold and underlined, respectively. To account for performance variability across different scenes, the results for LFAMF are reported as Mean ± Standard Deviation (SD). Furthermore, a paired t-test was conducted between LFAMF and the second-best method across all test scenes, yielding p < 0.05 for all datasets, which confirms that the performance improvements are statistically significant. As shown in the table, LFAMF consistently outperforms all other methods in terms of average PSNR and SSIM across all five datasets.

**Table 2:** Quantitative comparisons of different methods on 2 × 2-7 × 7 task.

Method	HCInew	HCIold	30Scenes	Occlusions	Reflective	Average
ShearedEPI [26]	31.84/0.898	37.61/0.942	39.17/0.975	34.41/0.955	36.38/0.944	35.88/0.943
Kalantari [7]	32.85/0.909	38.58/0.944	41.40/0.982	37.25/0.972	38.09/0.953	37.63/0.952
Yeung [27]	32.30/0.900	39.69/0.941	42.77/0.986	38.88/0.980	38.33/0.960	38.39/0.953
Liu [4]	32.81/0.906	39.72/0.944	42.71/0.985	39.07/0.980	38.53/0.960	38.57/0.955
SADenseNet [6]	33.19/0.911	39.85/0.946	43.06/0.986	39.48/0.981	38.99/0.960	38.91/0.957
LFASR-geo [8]	34.60/0.937	40.84/0.960	42.53/0.985	38.36/0.977	38.20/0.955	38.91/0.963
GAM [17]	34.31/0.935	40.84/0.954	42.91/0.987	39.06/0.981	39.04/0.962	39.23/0.964
LFRTR [33]	35.32/0.944	41.11/0.956	43.58/0.988	**40.22**/0.984	**39.76**/0.964	40.00/0.967
DistgASR [32]	34.70/0.974	**42.18/0.978**	43.67/0.995	39.46/0.991	39.11/0.978	39.82/0.983
LFAMF	**36.25 ± 0.82/ 0.978 ± 0.011**	41.73 ± 1.24/ 0.971 ± 0.015	**43.71 ± 0.95/ 0.995 ± 0.004**	40.08 ± 1.12/ **0.992 ± 0.006**	39.52 ± 1.08/ **0.980 ± 0.010**	**40.26 ± 1.04/ 0.983 ± 0.009**

^a^Metrics for the proposed LFAMF are presented as Mean ± Standard Deviation (SD). To assess statistical significance, paired t-tests were conducted between LFAMF and the second-best performing method across all scenes. The resulting p-values for both PSNR and SSIM are less than 0.05, indicating that the performance improvements are statistically significant.

Among the competitors, the EPI-based ShearedEPI [26] yields the lowest performance. This is primarily because it formulates reconstruction as the fusion of sheared EPIs and performs upsampling strictly within the EPI domain, thereby neglecting crucial spatial contextual information. Compared with the three non-depth-based methods (Yeung [27], Liu [4], and SADenseNet [6]), LFAMF achieves significant average PSNR gains of 1.87 dB, 1.69 dB, and 1.35 dB, respectively. Notably, the superiority of LFAMF is more pronounced on the HCInew and HCIold datasets, which feature large disparities. This demonstrates that the synergistic integration of multi-dimensional LF features and explicit geometric priors effectively enhances the model’s ability to handle large-disparity scenarios.

Furthermore, compared with depth-based methods (Kalantari [7], LFASR-geo [8], and GAM [17]), LFAMF exhibits average PSNR improvements of 2.19 dB, 1.41 dB, and 0.77 dB on the three real-world datasets (30Scenes, Occlusions, and Reflective). The performance gap stems from the fact that Kalantari [7] and LFASR-geo [8] rely solely on geometric consistency, which often fails in real-world scenes with small disparities or complex light transport. While GAM [17] incorporates multi-dimensional features, LFAMF still surpasses it by 1.03 dB/0.019 in average PSNR/SSIM.

Benefiting from the self-attention mechanism, the LFRTR [33] model effectively models long-range dependencies, showing clear advantages over other CNN-based methods, particularly on the Occlusions and Reflective datasets. The current leading method, DistgASR [32], employs a disentanglement mechanism to extract features from the spatial, angular, and EPI domains, achieving high reconstruction accuracy. Nevertheless, LFAMF outperforms DistgASR [32] by an average margin of 0.44 dB. The performance gain can be attributed to the fact that LFAMF not only aggregates intrinsic multi-dimensional features but also introduces an explicit geometric prior. The attention-guided fusion of these two streams allows the network to leverage their complementary strengths, resulting in superior reconstruction fidelity.

**Fig 9:**
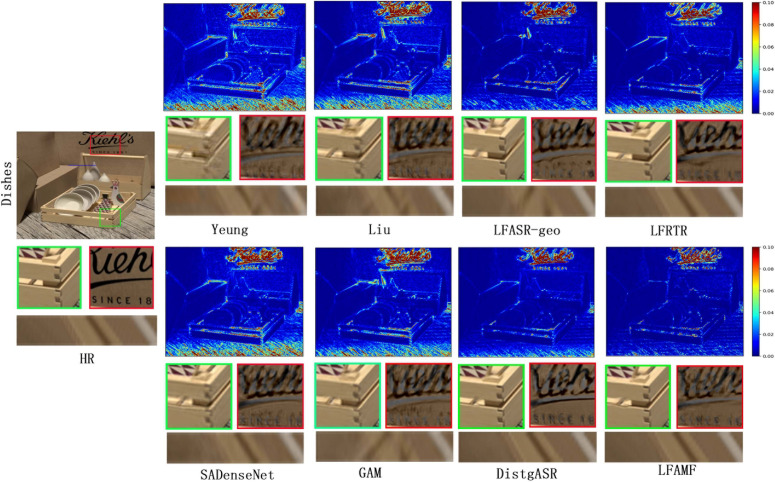
Visual comparisons of different methods on synthetic datasets [38] for 2×2-7×7 task. (All results are rendered by the authors under the same CC BY license mentioned in Fig 1).

**Fig 10:**
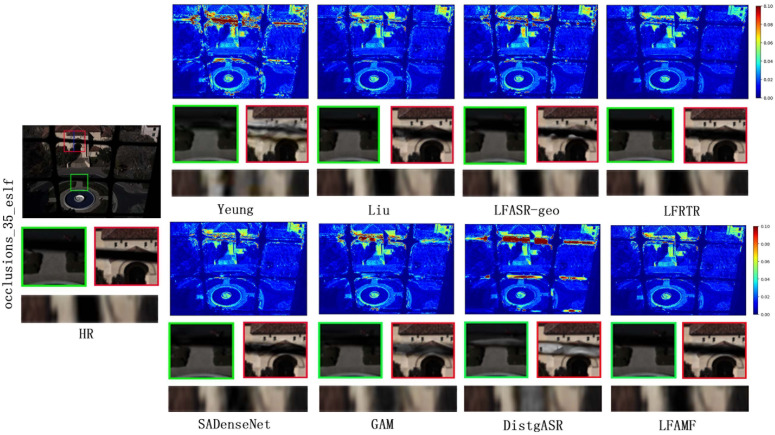
Visual comparisons of different methods on real-world datasets [37] for 2×2-7×7 task. (All results are rendered by the authors under the same CC BY license mentioned in Fig 1).

2 *Qualitative results*. Fig 9 and 10 present the visual reconstruction results on synthetic and real-world datasets, respectively. These visualizations include the central view of the high-resolution (HR) LF, error maps, Epipolar Plane Images (EPIs), and close-up views of representative local regions.

(1) *Synthetic scene analysis*. In the *Dishes* scene (Fig 9), several challenging details, such as alphanumeric characters, wood grain textures, and sharp box edges, are difficult to recover. As indicated by the red boxes, most competing methods struggle to reconstruct the structural integrity of the letters on the wall. Specifically, Yeung [27], Liu [4], LFRTR [33], SADenseNet [6], and GAM [17] exhibit significant errors in the floor texture. In contrast, LFASR-geo [8] yields relatively fewer errors in texture regions, as its depth-based approach can effectively project complex textures to novel views via depth information. However, as shown in the green boxes, methods like Yeung [27], Liu [4], LFASR-geo [8], SADenseNet [6], and GAM [17] suffer from noticeable artifacts at the sharp edges of the box. Benefiting from the self-attention mechanism, LFRTR [33] produces clearer edge structures. DistgASR [32] leverages a disentanglement mechanism to extract features from spatial, angular, and EPI domains, resulting in fewer errors in wood textures and box edges compared to other baselines. Remarkably, LFAMF achieves the highest fidelity, with the least error in both complex textures and geometric boundaries, closely approximating the ground truth. This demonstrates that the synergistic integration of multi-dimensional features and geometric priors in LFAMF significantly boosts reconstruction performance.
(2) *Real-world scene analysis*. Fig 10 depicts the occlusion_35_eslf scene, where background buildings and trees are occluded by a foreground window frame. As highlighted in the red and green boxes, Yeung [27], GAM [17] and DistgASR [32] produce “holes” or voids in the window frame, while Liu [4], LFASR-geo [8], and SADenseNet [6] exhibit varying degrees of ghosting artifacts at occlusion boundaries. Thanks to the global modeling capability of self-attention, LFRTR [33] captures more comprehensive intra- and inter-view complementary information, leading to fewer artifacts. Nevertheless, LFAMF reconstructs the most complete window frame with the minimal amount of artifacts. The error maps further confirm that our model generates the lowest pixel-wise error in both textured and occluded regions.
(3) *EPI consistency analysis*. Fig 9 and 10 also compare the EPIs generated by different methods. In the *Dishes* scene, the EPIs produced by Yeung [27], Liu [4], LFASR-geo [8], and GAM [17] show obvious misalignment and distortions, while SADenseNet [6] yields blurry results. In contrast, LFRTR [33], DistgASR [32], and our LFAMF maintain clear and consistent linear structures. In the occlusion_35_eslf scene, the EPIs of Yeung [27] and DistgASR [32] exhibit discontinuities, and the depth-based methods (GAM [17] and LFASR-geo [8]) suffer from severe artifacts. Liu [4] and SADenseNet [6] also result in varying levels of blurring. Conversely, the EPIs reconstructed by LFAMF are the sharpest and most consistent with the ground truth. These comparisons highlight that LFAMF is superior in preserving the inherent disparity structure of the LF.

#### Evaluation on 2 × 2–8 × 8 reconstruction.

1 *Quantitative evaluation*. The 2×2–8×8 reconstruction task is significantly more challenging due to the substantially increased disparity between the input views. As summarized in Table 3, the quantitative results exhibit a trend consistent with the 2×2–7×7 task. To ensure a robust evaluation, the results for LFAMF are reported as Mean ± SD, and paired t-tests confirm that its improvements over the second-best methods are statistically significant (p < 0.05). As shown, LFAMF achieves the highest average PSNR and SSIM scores across both synthetic and real-world datasets.

**Table 3:** Quantitative comparisons of different methods on 2 × 2-8 × 8 task.

Method	HCInew	HCIold	30Scenes	Occlusions	Reflective	Average
ShearedEPI [26]	26.50/0.775	35.99/0.902	36.74/0.950	32.98/0.943	34.38/0.941	32.96/0.892
Kalantari [7]	32.37/0.905	38.22/0.944	39.88/0.979	35.34/0.962	34.99/0.940	36.02/0.943
Yeung [27]	32.07/0.895	37.44/0.927	40.67/0.979	36.24/0.977	35.72/0.945	36.15/0.939
Liu [4]	32.09/0.892	38.75/0.938	41.61/0.983	37.28/0.973	36.16/0.950	37.18/0.947
SADenseNet [6]	31.38/0.875	38.06/0.931	41.46/0.982	37.10/.973	35.85/0.946	36.76/0.941
LFASR-geo [8]	33.78/0.932	40.48/0.953	41.70/0.984	36.79/0.972	36.05/0.950	37.76/0.958
LFRTR [33]	33.03/0.914	40.11/0.947	42.39/0.986	37.95/0.978	36.48/0.951	37.99/0.955
DistgASR [32]	33.94/0.964	**41.57/0.972**	42.72/0.994	38.12/0.988	36.54/0.972	38.58/0.978
LFAMF	**34.95 ± 0.78/ 0.971 ± 0.013**	41.47 ± 1.35/ 0.971 ± 0.016	**42.94 ± 1.02/ 0.994 ± 0.005**	**38.36 ± 1.15/ 0.989 ± 0.007**	**36.93 ± 1.21/ 0.974 ± 0.012**	**38.93 ± 1.10/ 0.980 ± 0.011**

^a^ Metrics for the proposed LFAMF are presented as Mean ± Standard Deviation (SD). To assess statistical significance, paired t-tests were conducted between LFAMF and the second-best performing method across all scenes. The resulting p-values for both PSNR and SSIM are less than 0.05, indicating that the performance improvements are statistically significant.

Specifically, the performance gap for the EPI-based ShearedEPI [26] becomes even more pronounced, with its average PSNR trailing behind the early Kalantari [7] method by 3.06 dB. Compared to the three non-depth-based methods (Yeung [27], Liu [4], and SADenseNet [6]), LFAMF yields substantial average PSNR gains of 2.78 dB, 1.75 dB, and 2.17 dB, respectively. It is noteworthy that Liu [4] outperforms SADenseNet [6] in this 8×8 task, which can be attributed to its effective integration of epipolar information from four angular directions.

When compared with depth-based methods, LFAMF surpasses Kalantari [7] and LFASR-geo [8] by 2.91 dB and 1.17 dB in average PSNR. The limited accuracy of depth estimation on small-disparity real-world datasets hampers the performance of LFASR-geo [8]. While the LFRTR [33] model demonstrates clear advantages over LFASR-geo [8], Liu [4], and SADenseNet [6] in terms of average metrics due to its global self-attention modeling, its performance on the large-disparity HCInew and HCIold datasets falls below that of LFASR-geo [8]. This is because the input views for the 2×2–8×8 task are extremely sparse, making it difficult for LFRTR [33] to capture the underlying geometric structure solely from the spatial and angular domains. In contrast, LFAMF outperforms the current state-of-the-art DistgASR [32] by 0.35 dB in average PSNR. This performance gain, supported by high statistical confidence (p < 0.05), further validates the superiority and robustness of our proposed framework under extreme angular sparsity.

**Fig 11:**
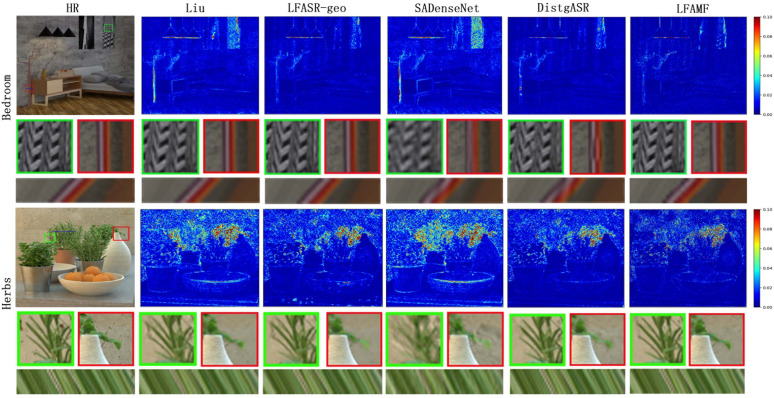
Visual comparisons of different methods on synthetic datasets [38] for 2×2-8×8 task. (All results are rendered by the authors under the same CC BY license mentioned in Fig 1).

**Fig 12:**
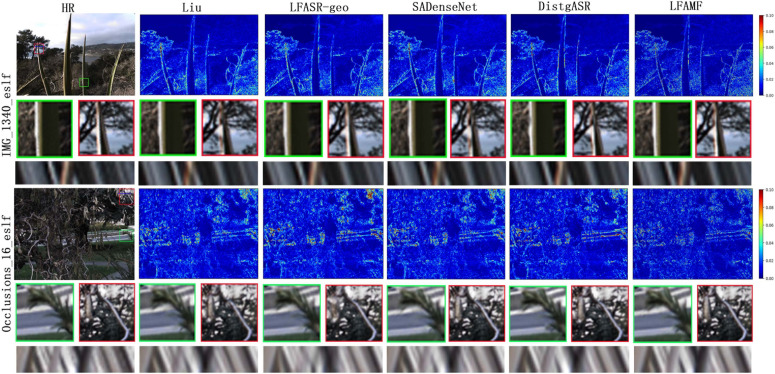
Visual comparisons of different methods on real-world datasets [37] for 2×2-8×8 task. (All results are rendered by the authors under the same CC BY license mentioned in Fig 1).

2 *Qualitative results*. Fig 11 and 12 illustrate the visual reconstruction results of all competing methods on synthetic and real-world datasets, encompassing challenging scenarios such as occlusions, complex textures, and fine structures.

(1) *Synthetic scene analysis*. In the synthetic datasets shown in Fig 11, the large disparity range makes it difficult for most LF reconstruction methods to accurately capture geometric information. Methods like Liu [4] and SADenseNet [6] fail to model depth information during reconstruction, resulting in inferior performance compared to LFAMF. In the *Bedroom* scene, these two methods exhibit varying degrees of blurring on the wall textures and severe artifacts along the clothes hanger edges. Although the depth-based LFASR-geo [8] recovers the wall texture better by utilizing geometric priors, it still struggles with structural integrity. As highlighted in the red box, the clothes hanger reconstructed by DistgASR [32] suffers from noticeable distortion. In the *Herbs* scene, SADenseNet [6] produces extremely blurry results where the leaf structures are nearly unrecognizable. Similarly, Liu [4] exhibits blurring at the leaf boundaries and prominent artifacts at the fruit plate edges. While LFASR-geo [8] visually outperforms the former two, it still yields significant errors in the table texture and plate boundaries. DistgASR [32], which fuses spatial, angular, and EPI features via a disentanglement mechanism, provides clearer results. In contrast, LFAMF synthesizes significantly sharper details across both complex textures and occlusion boundaries.
(2) *Real-world scene analysis*. The real-world datasets in Fig 12 involve natural lighting, occlusions, and reflective surfaces. In the IMG_1340_eslf scene, all competing methods show substantial artifacts at the branch edges (see red and green boxes), primarily due to their failure to fully exploit multi-dimensional features and geometric structures. Similar observations can be made in the Occlusions_16_eslf scene, where LFASR-geo [8] generates the largest reconstruction errors in dense branch occlusions and road areas. Liu [4], SADenseNet [6], and DistgASR [32] all exhibit varying levels of blurring and ghosting at the branch boundaries. Conversely, LFAMF effectively suppresses ghosting and blurring, better recovering fine edge details.
(3) *EPI and disparity structure consistency*. The LF disparity structure implicitly represents the scene’s geometry and is the most valuable information in an LF image. As shown in the EPI comparisons in Figs. 11 and 12, Liu [4] and SADenseNet [6] fail to capture long-range dependencies in large-disparity and occluded scenes due to their limited receptive fields, resulting in disordered and blurry EPIs (e.g., *Bedroom*, *Herb*s, and IMG_1340_eslf). In real-world scenes with small disparities, LFASR-geo [8] struggles to estimate precise disparity values, leading to discontinuities at occlusion boundaries (e.g., Occlusions_16_eslf). While DistgASR [32] performs better than the other three, it still shows obvious deformation in the *Bedroom* scene. By directly upsampling in the angular domain to synthesize novel views, LFAMF thoroughly explores inter-view correlations, yielding clearer EPI textures and superior preservation of the LF disparity structure.

#### Computational efficiency and practical utility.

As summarized in Table 4, we provide a comprehensive analysis of the proposed LFAMF alongside several representative methods. It is observed that LFAMF entails a higher parameter count and increased computational requirements (Runtime and Memory) compared to some lightweight models like DistgASR [32]. This is primarily attributed to our dual-branch synergy and the integration of 3D convolutions, which are essential for capturing complex spat-angular dependencies and ensuring robustness in challenging scenarios. However, we argue that these costs are well within an acceptable and comparable range for modern high-performance computing environments. Specifically, even for the most demanding 2×2–8×8 task, LFAMF maintains an inference speed of 3.28s per LF and a memory footprint of 17583MB on an NVIDIA RTX 3090 Ti. Considering the significant performance gains in reconstruction accuracy (as shown in Tables 2 and 3), our model offers a superior performance-efficiency trade-off. The additional complexity is a justified investment for achieving state-of-the-art visual quality and structural consistency in complex LF scenes.

**Table 4:** Computational efficiency and resource consumption of different methods.

Scale	Method	#Params.	FLOPs	Runtime	Memory	PSNR/SSIM
2×2–7×7	Liu [4]	1.19M	21.11G	2.53s	10142M	38.57/0.955
	SADenseNet [6]	1.09M	39.76G	2.46s	10846M	38.91/0.957
	LFASR-geo [8]	1.01M	162.46G	2.15s	12440M	38.91/0.963
	LFRTR [33]	2.15M	70.37G	2.13s	17351M	40.00/0.967
	DistgASR [32]	2.68M	156.77G	2.37s	15146M	39.82/0.983
	LFAMF	6.27M	107.23G	3.16s	16832M	**40.26/0.983**
2×2–8×8	Liu [4]	2.01M	35.84G	2.87s	12882M	37.18/0.947
	SADenseNet [6]	1.14M	41.34G	2.52s	22931M	36.76/0.941
	LFASR-geo [8]	1.11M	207.22G	2.26s	13352M	37.76/0.958
	LFRTR [33]	2.23M	90.45G	2.23s	19163M	37.99/0.955
	DistgASR [32]	2.74M	158.06G	2.41s	16891M	38.58/0.978
	LFAMF	6.04M	112.35G	3.28s	17583M	**38.93/0.980**

### Ablation study

The LFAMF model integrates three core components: MFNet, GSPNet, and AFNet. To evaluate the individual contribution of each subnetwork, we conducted a series of ablation experiments for the 2×2–7×7 task.

**Fig 13:**
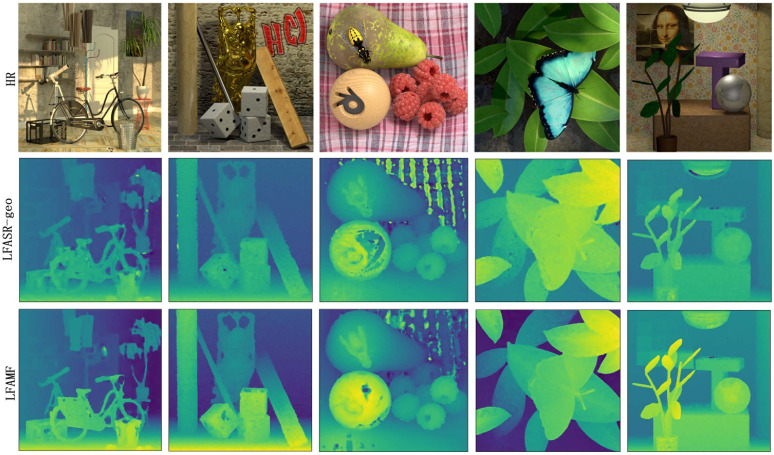
Comparison of depth maps generated by LFAMF and LFASR-geo [8] across different scenes from the HCInew [38] and HCIold [39] datasets. (All results are rendered by the authors under the same CC BY license mentioned in Fig 1).

**Fig 14:**
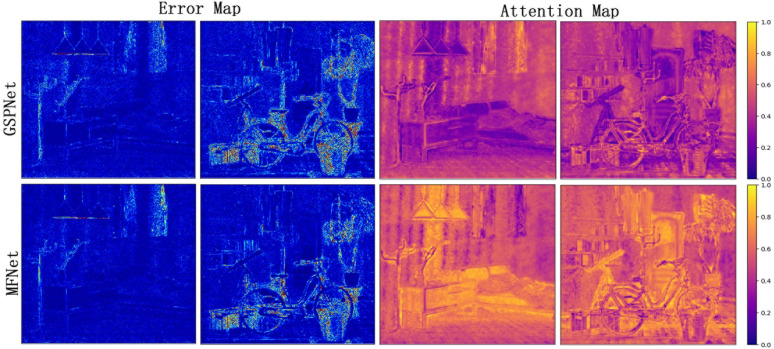
Visual comparisons of attention-based fusion in different scenes from the HCInew [38] dataset. (All results are rendered by the authors under the same CC BY license mentioned in Fig 1).

(1) *Necessity of the two-branch architecture*: To justify our dual-stream design, we first evaluated the performance of each branch independently. The “MFNet-only” variant, which combines SAEBlock and PVSBlock but lacks geometric guidance, achieves an average PSNR of 38.21 dB. Meanwhile, the “GSPNet-only” variant, relying exclusively on explicit geometric warping, yields only 37.95 dB. The full LFAMF model outperforms these single-branch baselines by 0.78 dB and 1.04 dB, respectively. This substantial gain confirms that neither implicit features nor explicit geometry alone can handle complex LF scenes, and their synergistic combination is essential for superior reconstruction.
(2) *Effectiveness of MFNet*. MFNet consists of two parallel branches: SAEBlock and PVSBlock. The former extracts subspace features from the spatial, angular, and EPI domains, while the latter treats LF sub-aperture images as a pseudo-video sequence to capture spatio-angular correlations. To verify the performance gain from these branches, we sequentially removed each branch and retrained the resulting variant models. As summarized in Table 5, the reconstruction performance declines to varying degrees upon the removal of either branch. Notably, the PVSBlock contributes more significantly to the overall reconstruction accuracy.
(3) *Effectiveness of GSPNet*. The GSPNet introduces explicit geometric priors via an encoder-decoder based depth estimation module. To validate its effectiveness, we first compared its estimated disparity maps with those of LFASR-geo [8]. Unlike our module, LFASR-geo [8] utilizes a series of cascaded 2D convolutions with large 7×7 kernels and dilated convolutions to expand the receptive field. Fig. 13 illustrates the disparity maps for five scenes estimated by both methods. It is evident that the disparity maps generated by LFAMF are more precise, with significantly fewer errors in background regions and along occlusion boundaries, demonstrating the superiority of our Encoder-Decoder architecture. Furthermore, removing GSPNet from the full model led to a drop in PSNR of 0.06 dB and 0.2 dB on the two datasets, respectively (see Table 5). This confirms that the geometric structure information extracted by GSPNet is vital for enhancing reconstruction quality.
(4) *Effectiveness of AFNet*. The AFNet is designed to adaptively fuse the intermediate results from MFNet and GSPNet. To assess its impact, we replaced AFNet with a standard attention module to directly merge the outputs of the two branches. As shown in Table 5, the variant model without AFNet suffered a performance decrease of 0.67 dB and 0.37 dB in PSNR on the HCInew and HCIold datasets, respectively. This indicates that AFNet effectively harmonizes the complementary information between intrinsic multi-dimensional features and geometric priors. Fig 14 provides a visualization of the error maps and the learned attention maps for the two subnetworks. The attention mechanism assigns lower weights to high-error regions and higher weights to high-fidelity areas, thereby leveraging the strengths of both streams to achieve superior reconstruction accuracy.
(5) *Effectiveness of* $L_{smooth}$. To further verify the efficacy of the geometric regularization, we investigate the impact of the smoothness loss $L_{smooth}$ by evaluating a baseline variant trained with λ=0. As presented in Table 5, excluding $L_{smooth}$ results in a performance degradation, with the average PSNR dropping from 38.99 dB to 38.85 dB on the two datasets. This outcome demonstrates that the edge-preserving smoothness constraint is indispensable for regularizing scene disparity fields, maintaining sub-pixel angular consistency, and securing high structural fidelity in the final synthesized LF views.

**Table 5:** Effectiveness analysis results.

Model					HCInew	HCIold	Average
SAEBlock	PVSBlock	GSPNet	AFNet	$L_{smooth}$			
	✓	✓	✓	✓	36.00/0.977	41.68/0.969	38.84/0.973
✓		✓	✓	✓	35.25/0.971	41.60/0.973	38.43/0.972
✓	✓		✓	✓	36.19/0.976	41.53/0.970	38.86/0.973
✓	✓			✓	35.12/0.965	41.29/0.969	38.21/0.967
		✓		✓	34.87/0.957	41.02/0.961	37.95/0.959
✓	✓	✓		✓	35.58/0.973	41.36/0.969	38.47/0.971
✓	✓	✓	✓		36.12/0.972	41.58/0.964	38.85/0.968
✓	✓	✓	✓	✓	**36.25/0.978**	**41.73/0.971**	**38.99/0.975**

### Limitation analysis

**Fig 15:**
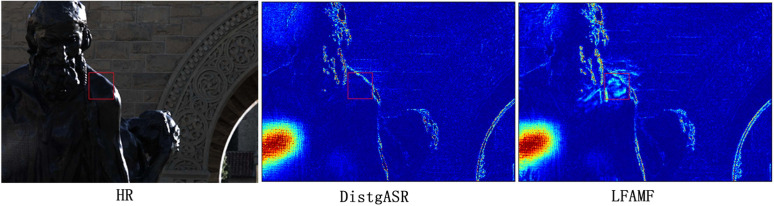
Analysis of a representative failure case on the Reflective [37] dataset. (All results are rendered by the authors under the same CC BY license mentioned in Fig 1).

Despite the superior average performance, it is essential to acknowledge the limitations of LFAMF. Among the 15 reflective scenes tested, our method failed to outperform all competitors in 3 instances, primarily those featuring complex specular reflections. Fig 15 illustrates a representative failure case. Compared to DistgASR [32], which employs a feature disentanglement mechanism, LFAMF exhibits higher pixel-wise errors in the highlighted specular region (red box). The diagnosis suggests that in scenarios with extreme non-Lambertian properties, the explicit geometric priors generated by our GSPNet may encounter ambiguity, as the constant brightness assumption is violated. Although the AFNet attempts to balance the two branches, the reliance on potentially inaccurate geometric warping can still introduce artifacts that are less prevalent in purely feature-based methods like DistgASR. This reveals that while our hybrid architecture is robust for most cases, its performance under extreme light transport conditions (e.g., strong specularities or transparency) warrants further optimization in future work.

## Conclusion

This paper proposes the light field attention-guided multi-dimensional fusion network (LFAMF) to address the challenges of angular super-resolution in complex scenes with severe occlusions and large disparities. By synergistically integrating multi-dimensional features from the spatial, angular, EPI, and pseudo-video sequence domains with explicit geometric priors, the LFAMF effectively overcomes the limitations of existing methods that rely on single-dimensional representations. Experimental results on various datasets indicate that the proposed model not only achieves superior quantitative performance but also significantly improves reconstruction quality in occluded and highly textured areas. Notably, the use of sub-pixel and transposed convolutions ensures the preservation of LF angular consistency. Overall, the LFAMF demonstrates strong robustness and fidelity, making it a promising approach for practical LF imaging applications.
